# Transcranial static magnetic stimulation over the motor cortex can facilitate the contralateral cortical excitability in human

**DOI:** 10.1038/s41598-021-84823-4

**Published:** 2021-03-08

**Authors:** Yasuyuki Takamatsu, Satoko Koganemaru, Tatsunori Watanabe, Sumiya Shibata, Yoshihiro Yukawa, Masatoshi Minakuchi, Ryota Shimomura, Tatsuya Mima

**Affiliations:** 1 Department of Rehabilitation Science, Faculty of Health Sciences, Hokkaido University, Sapporo, 060-0812 Japan; 2 Kinugasa Research Organization, Ritsumeikan University, Kyoto, 603-8577 Japan; 3 Department of Physiology, Dokkyo Medical University, Shimotsugagun, 321-0293 Japan; 4 Department of Sensorimotor Neuroscience, Graduate School of Biomedical and Health Sciences, Hiroshima University, Hiroshima, 734-8553 Japan; 5 Department of Rehabilitation, Murata Hospital, Osaka, 544-0011 Japan; 6 Graduate School of Core Ethics and Frontier Sciences, Ritsumeikan University, Kyoto, 603-8577 Japan

**Keywords:** Neuroscience, Neurology

## Abstract

Transcranial static magnetic stimulation (tSMS) has been focused as a new non-invasive brain stimulation, which can suppress the human cortical excitability just below the magnet. However, the non-regional effects of tSMS via brain network have been rarely studied so far. We investigated whether tSMS over the left primary motor cortex (M1) can facilitate the right M1 in healthy subjects, based on the hypothesis that the functional suppression of M1 can cause the paradoxical functional facilitation of the contralateral M1 via the reduction of interhemispheric inhibition (IHI) between the bilateral M1. This study was double-blind crossover trial. We measured the corticospinal excitability in both M1 and IHI from the left to right M1 by recording motor evoked potentials from first dorsal interosseous muscles using single-pulse and paired-pulse transcranial magnetic stimulation before and after the tSMS intervention for 30 min. We found that the corticospinal excitability of the left M1 decreased, while that of the right M1 increased after tSMS. Moreover, the evaluation of IHI revealed the reduced inhibition from the left to the right M1. Our findings provide new insights on the mechanistic understanding of neuromodulatory effects of tSMS in human.

## Introduction

Human brain is a dynamic network connecting multiple regions with different functions into a whole. As a result of this network property, a local functional change in one brain region can produce the functional alteration of the remote region, if two areas are tightly connected anatomically and/or functionally. One simplified example is the interhemispheric connection in which the homologous areas of both hemispheres are coupled to balance the competition and cooperation between them.

Due to the predominance of inhibitory function in this interhemispheric connection (interhemispheric inhibition: IHI), it has been reported that the suppression of one brain region can cause the functional facilitation of the contralateral region, via the reduction of IHI from the suppressed site to the other side^1–3^. This phenomenon is called paradoxical functional facilitation (PFF)^4^, which can be found in visual, attention and motor functions across experimental animal and human studies^5–9^. Functional suppression of the healthy hemisphere by using repetitive transcranial magnetic stimulation (rTMS) has been clinically applied for rehabilitation purposes to facilitate the lateralized affected motor^10^ and non-motor^11, 12^ functions in stroke.

Recently, for suppressing human local brain function noninvasively, transcranial static magnetic stimulation (tSMS) has been introduced, where a strong compact permanent magnet was placed over the scalp to modulate the brain function just below^13–22^. Although its physiological mechanism has not been fully clarified, it is likely that the neural suppressions might be related to the functional change of ion channels by magnetic torque^23^.

Thus, to investigate the remote effect of the focal brain modulation through neural network, we tested the motor excitability of the right M1 after tSMS intervention on the left M1, based on the hypotheses that the suppressions of M1 would produce the PFF and facilitate the motor function of the right M1 (Experiment 1) and that this remote effect is associated with the reduction of the IHI from the left to the right M1 (Experiment 2).

To estimate the motor excitability, the amplitude of the motor evoked potentials (MEPs) from a corresponding muscle induced by TMS over the contralateral M1 was used^24^. To evaluate IHI, paired-pulse TMS over each M1 with a short delay (10 and 40 ms) was used. The amplitude of MEPs induced by the latter stimulus (test stimulus) is reduced compared to that of the single-pulse TMS, due to the conditioning effects of the former stimulus (conditioning stimulus) over another M1^1^. The IHI is defined as the ratio between the amplitude of the paired test MEP and that of the single MEP. The greater the decrease of MEPs on test stimulus is, the greater the IHI is.

## Results

**Figure 1:**
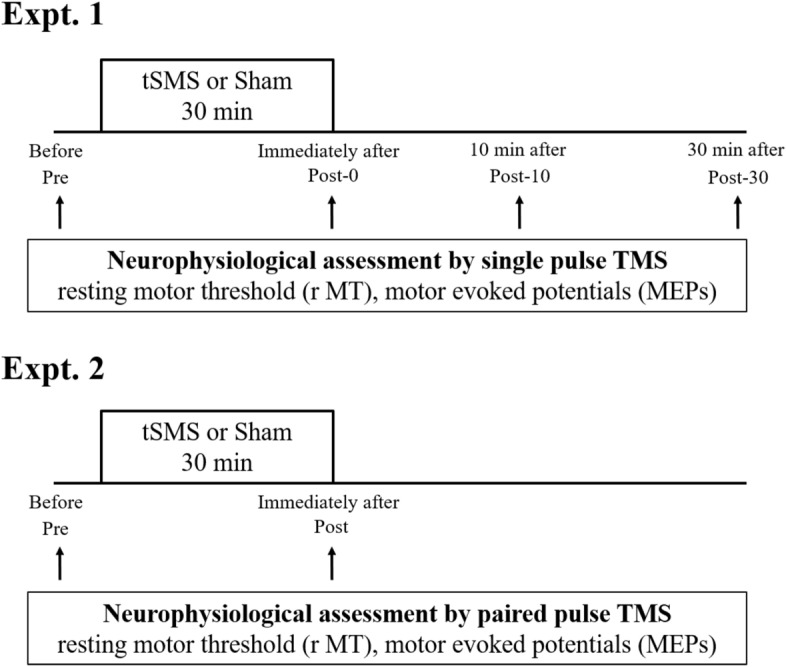
Experimental procedures. TSMS or sham stimulation was applied over the left M1 for 30 min in Expt. 1 and 2. For Expt. 1, neurophysiological assessment by single-pulse TMS was performed at before (pre), immediately after (Post-0), 10 min (Post-10) and 30 min (Post-30) after tSMS or sham stimulation. At each point, rMT and MEP were measured from the both hands respectively. For Expt. 2, we measured the IHI using paired-pulse TMS before (Pre) and immediately after (Post) tSMS or sham stimulation.

Figure 1 shows the experimental procedures in this study. There were no adverse events for all subjects during or after tSMS and transcranial magnetic stimulation (TMS) measurement procedure.

### Experiment 1: the effects of tSMS on cortical excitability in the both hemispheres

To reveal the effect of unilateral tSMS on ipsilateral as well as contralateral M1 (remote region), we measured the MEPs from the both first dorsal interosseous (FDI) muscles to assess the corticospinal excitability, after tSMS or non-magnetic sham intervention over the left M1.

Table 1 shows the summary of mean ± SEM of resting motor threshold (rMT) (machine output intensity), SI1mV and MEPs peak-to-peak amplitude in real and sham stimulation. For rMT, three-way repeated-measures ANOVA (Time × Hands × Condition) showed no significant interactions (F_3, 54_ = 2.053, p = 0.117, ηp^2^ = 0.10). For SI1mV, two-way ANOVA did not show the Hand × Condition interaction (F_1, 18_ = 1.558, p = 0.228, ηp^2^ = 0.08).

**Table 1:** The summary of results in Experimental 1.

	Real stimulation		Sham stimulation	
	Left hand	Right hand	Left hand	Right hand
**rMT (%)**				
Pre	46 ± 3	45 ± 3	42 ± 2	43 ± 2
Post-0	46 ± 3	45 ± 3	42 ± 2	43 ± 2
Post-10	46 ± 3	46 ± 3	42 ± 2	44 ± 2
Post-30	47 ± 3	45 ± 3	42 ± 2	44 ± 2
SI1mV (%)	59 ± 3	57 ± 3	54 ± 3	56 ± 3
**MEPs peak-to-peak amplitude (μV)**				
Pre	934 ± 106	1043 ± 89	870 ± 92	753 ± 49
Post-0	1093 ± 141	745 ± 81	756 ± 65	859 ± 83
Post-10	1032 ± 146	758 ± 85	858 ± 104	734 ± 75
Post-30	1089 ± 170	768 ± 87	756 ± 96	836 ± 87

Data are shown as mean ± SEM.

*rMT* resting motor threshold, *MEPs* motor evoked potentials, *SI1mV* intensity to obtain a 1 mV MEPS peak-to-peak amplitude.

**Figure 2:**
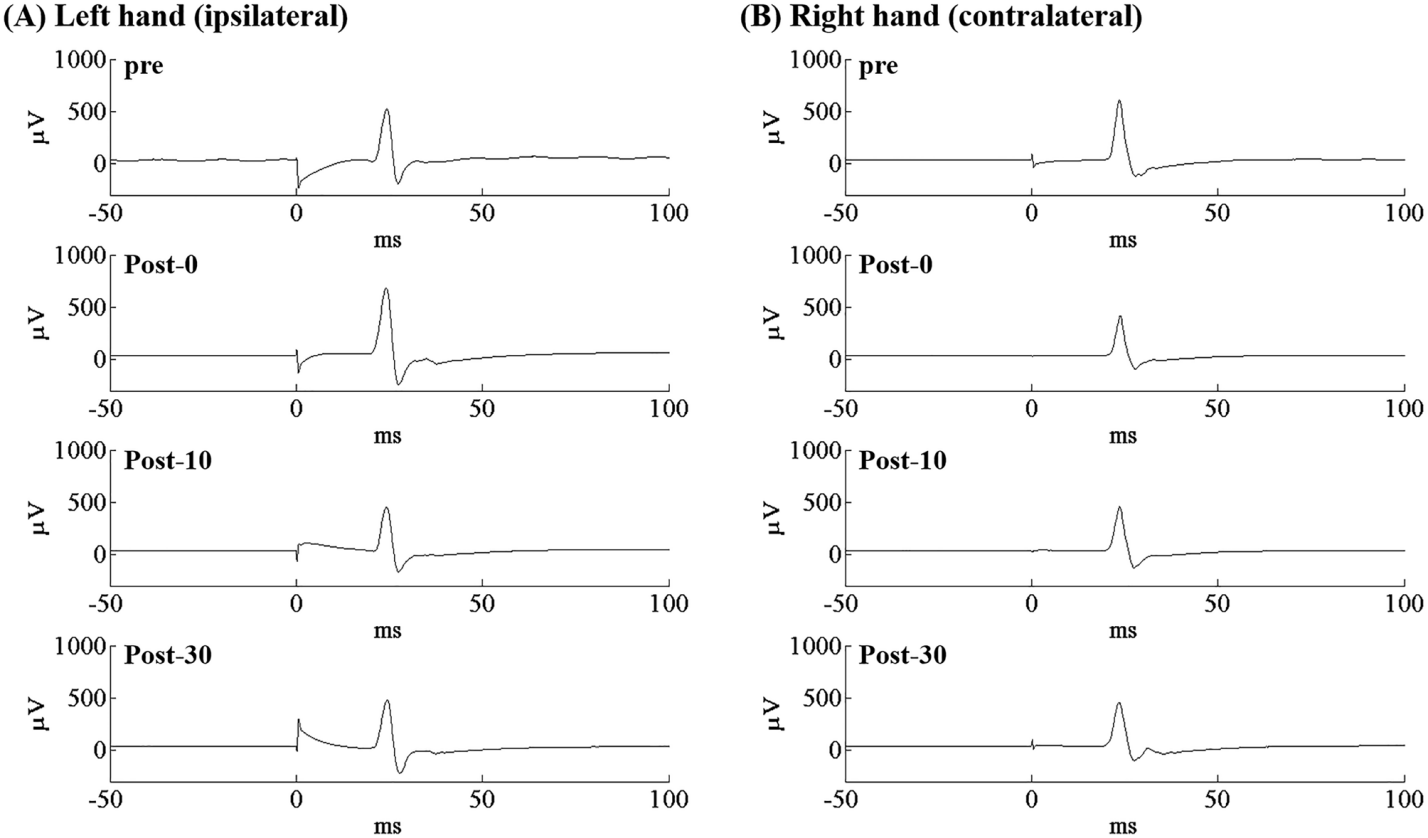
MEPs changes by the real stimulation in a representative subject. This figure shows MEPs changes in the left (**A**) and right (**B**) hand from a representative subject after the real stimulation. MEPs amplitude increases in the left hand immediately after the stimulation (**A**). On the other hand, it reduces in the right hand for at least 30 min after the stimulation (**B**).

**Figure 3:**
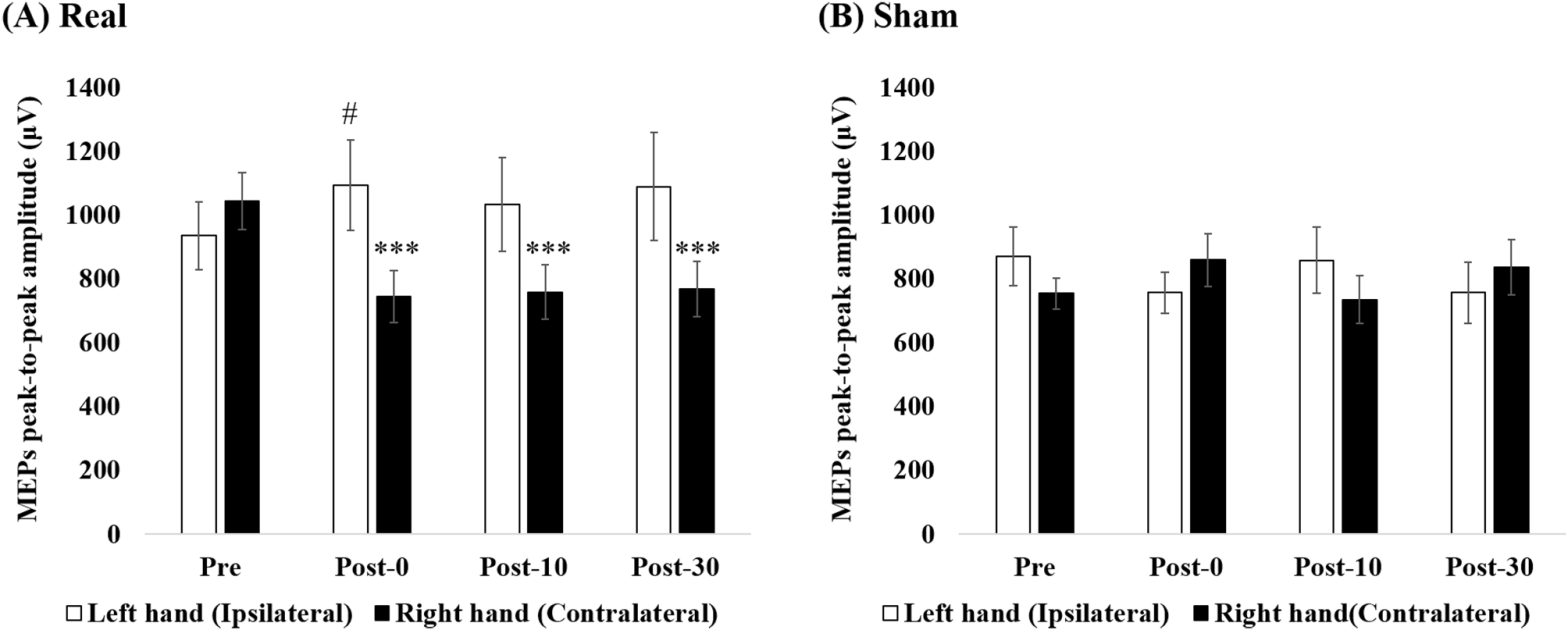
Effects of tSMS on the MEPs peak-to-peak amplitude in the right and left FDI muscle. Time × Hands × Group interaction was significant. When analyzed separately for real (**A**) and sham (**B**) tSMS conditions by two-way repeated-measures ANOVA, Time × Hands interactions was detected only for the real condition. On the other hands, there was no significant Time × Hands interactions in the sham condition. In the real condition, post hoc analysis showed that MEPs peak-to-peak amplitude was significantly decreased at Post-0, 10, and 30 compared to at Pre in the right hand (***p < 0.005 vs Pre). In the left hand, it was significantly increased at Post-0 compared to at Pre (^#^p < 0.05 vs Pre). Error bar are standard errors of the mean.

Figure 2 shows the MEPs waveforms for the real condition in a representative subject. Figure 3 shows the results of the mean MEPs peak-to-peak amplitude in real and sham condition. For MEPs, Time (Pre, Post-0, Post-10, Post-30) × Hands (right, left) × Condition (real, sham) interaction was significant (F_2.117, 38.105_ = 10.136, p < 0.001, ηp^2^ = 0.36). When analyzed separately for real and sham conditions by two-way repeated-measures ANOVA, Time × Hands interactions was detected only for the real condition (F_2.035, 36.634_ = 11.097, p < 0.001, ηp^2^ = 0.38, Fig. 3A). On the other hand, there was no significant Time × Hands interactions in the sham condition (F_3, 54_ = 2.603, p = 0.061, ηp^2^ = 0.13, Fig. 3B).

In the real condition, post hoc analysis showed that MEPs peak-to-peak amplitude was significantly decreased at Post-0, 10, and 30 compared to at Pre in the right hand (Post-0 vs Pre: p = 0.001, Cohen’s d = 0.77, Post-10 vs Pre: p = 0.003, Cohen’s d = 0.72, Post-30 vs Pre: p = 0.002, Cohen’s d = 0.69, Fig. 3A). In the left hand, it was significantly increased at Post-0 compared to at Pre (p = 0.030, Cohen’s d = 0.28, Fig. 3A).

### Experiment 2: the effects of tSMS on IHI from the left to the right hemisphere

By using paired-pulse TMS over each hemisphere with short delays (10 and 40 ms), IHI from the left to the right M1 can be measured. For Experiments 2, we hypothesized that tSMS would decrease IHI as well as the left M1 excitability, and measured IHI after tSMS or sham intervention.

Table 2 shows the summary of mean ± SEM of rMT for the right hand, SI1mV for the left hand and MEPs peak-to-peak amplitudes in Real and Sham stimulation. There were no significant differences between Condition in the rMT (p = 0.878) and SI1mV (p = 0.715).

**Table 2:** The summary of results in Experimental 2.

	Real stimulation			Sham stimulation		
rMT (%)	57 ± 3			57 ± 3		
SI1mV (%)	69 ± 4			67 ± 4		

MEPs peak-to-peak amplitude (μV)	Test	IHI10	IHI40	Test	IHI10	IHI40
Pre	1437 ± 212	930 ± 169	1134 ± 208	1401 ± 221	987 ± 182	944 ± 163
Post	1588 ± 236	1154 ± 238	1505 ± 280	1467 ± 268	935 ± 271	1101 ± 295

IHI ratio	IHI10		IHI40	IHI10		IHI40
Pre	0.63 ± 0.05		0.77 ± 0.06	0.68 ± 0.05		0.65 ± 0.05
Post	0.67 ± 0.05		0.92 ± 0.07	0.55 ± 0.05		0.69 ± 0.06

Data are shown as mean ± SEM.

*rMT* resting motor threshold, *MEPs* motor evoked potentials, *SI1mV* intensity to obtain a 1 mV MEPS peak-to-peak amplitude, *IHI* interhemispheric inhibition.

**Figure 4:**
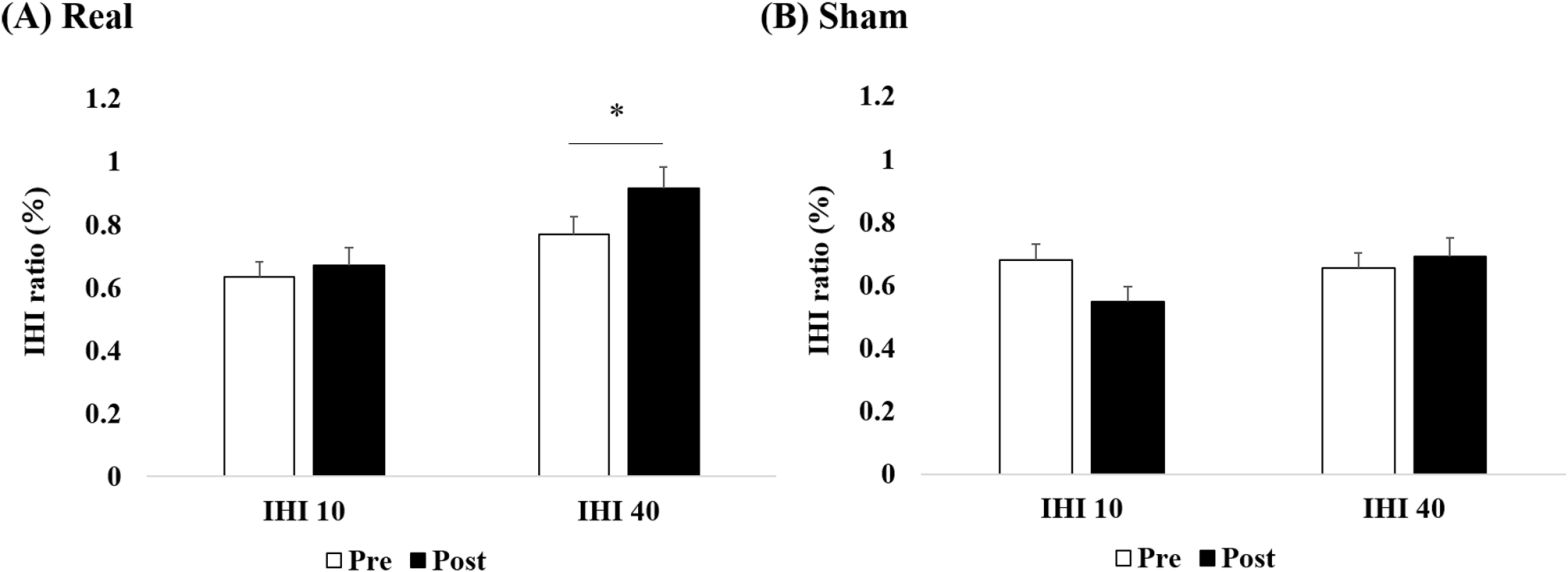
the effects of tSMS on IHI from the left to the right hemisphere. For real condition (**A**), the main effects of Time and IHI were significant. However, the interaction was not significant. Additional post-hoc t-test revealed the significant increase in IHI40 but not in IHI10 (*p < 0.05). For sham condition (**B**), no significant difference was found. Error bar are standard errors of the mean.

Figure 4 shows the results of IHI ratio in real and sham condition. Although three-way repeated-measures ANOVA (Time × ISI × Condition) showed no significant three-way interactions (F_1, 17_ = 0.302, p = 0.590, ηp^2^ = 0.02), two-factor interactions were all significant (Condition × Time: F_1, 17_ = 5.215, p = 0.036, ηp^2^ = 0.24, Condition × ISI: F_1, 17_ = 8.622, p = 0.009, ηp^2^ = 0.34, Time × ISI: F_1, 17_ = 7.728, p = 0.013, ηp^2^ = 0.31), suggesting the significant effect of Condition on Time and ISI. To exploratively evaluate the effect of real stimulation on IHI, we analyzed separately for real and sham conditions by two-way repeated-measures ANOVA (Time × ISI).

For real condition, the main effects of Time and ISI were significant (Time: F_1, 17_ = 6.159, p = 0.024, ηp^2^ = 0.27, ISI: F_1, 17_ = 9.093, p = 0.008, ηp^2^ = 0.35). However, the interaction was not significant (F_1, 17_ = 3.681, p = 0.072, ηp^2^ = 0.18). Additional post-hoc t-test revealed the significant increase in IHI40 (Pre vs Post: p = 0.034, Cohen’s d = 0.57) but not in IHI10 (Fig. 4.A). For sham condition, no significant difference was found (Time: F_1, 17_ = 1.219, p = 0.285, ηp^2^ = 0.07, ISI: F_1, 17_ = 1.435, p = 0.247, ηp^2^ = 0.08, Time × ISI: F_1, 17_ = 3.657, p = 0.073, ηp^2^ = 0.18, Fig. 4.B).

Additionally, the correlation (Spearman’s rho) between the excitability reduction of the left M1 and the reduction of IHI was not significant in real condition (IHI10: r = 0.125, p = 0.622, IHI40: r = -0.282, p = 0.257).

## Discussion

We investigated the effect of tSMS over the left M1 on the cortical excitability of the right M1 and found that the direction of MEPs change was inverse for the left and right hands in real stimulation (Expt. 1). Namely, the MEPs decreased in the right hand and increased in the left hand after tSMS, suggesting that the suppression of the cortical excitability in the unilateral hemisphere can induce the facilitation of the excitability in the contralateral hemisphere. Since a previous study reported that the MEP amplitude was not facilitated after tSMS of 20-min duration over the contralateral M1^25^, this transient facilitation effect in the contralateral hemisphere after 30-min intervention is a novel finding of this study. However, compared to the suppression effect directly induced by tSMS, the facilitation effect of contralateral M1 was only shown just after tSMS. Thus, it can be speculated that the longer and more stable facilitation in the contralateral non-stimulated M1 might be induced by the tSMS intervention with the duration of > 30 min.

Regarding the mechanism of this remote facilitation, we found that interhemispheric inhibition from the left to the right M1 just after the tSMS on the left M1 was significantly decreased as hypothesized (Expt. 2). Although further studies are necessary, it is likely that the reduced inhibition from the stimulated M1 (left) to non-stimulated one (right) might contribute to the increased excitability in the non-stimulated M1.

The suppression of the cortical excitability by tSMS on the stimulated M1 confirmed previous reports^13, 16, 20^, although the suppression effects of 30-min tSMS has been under ongoing discussion^26, 27^. Our results reproduced and confirmed that the 30-min tSMS have the suppression effects on corticospinal excitability as shown in previous study^20^. The longer duration of static magnetic fields showed the bigger effects on the decreasing of cortical excitability in an animal study^28^. Therefore, our 30-min tSMS showed the longer-lasting effect which was consistent with a previous study^20^ compared to tSMS studies with 10–20 min of duration^13, 16^. The mechanisms of suppression effect of tSMS on cortical excitability has been hypothesized that the magnetic torque induced the change of the morphology of the cell membrane lipid bilayer, and then ion channels was affected^23^. Indeed, it has been shown that static magnetic fields changed the function of some ion channels (e.g., sodium, potassium, calcium and chloride) in in-vitro study^29–34^. However, the physiologic mechanism of tSMS has not been clarified, and further animal studies are needed.

Regarding the IHI modulation caused by the suppression of M1 function, it was shown that there were two distinct phases of IHI from the unilateral M1 to contralateral one at ISIs of ~ 10 ms (short latency IHI) and ~ 50 ms (long latency IHI)^3^. The reduction of IHI from the stimulated to non-stimulated hemisphere has been reported for short latency IHI^35^ or both short and long latency IHI^36^, when the unilateral M1 was suppressed by the low-frequency rTMS. Cathodal transcranial direct current stimulation (tDCS) over the M1 also showed the reduction of IHI measured by ipsilateral silent period^37^. The functional facilitation of non-stimulated cortex has been found in the low-frequency rTMS study^35^, but not in the cathodal tDCS one^37^. In addition, transcranial alternating current stimulation (tACS) at beta frequency over the M1 did not induce any modulation of IHI^38^. It is likely that the different NIBS technique might be associated with the different pattern of modulation at the neuronal population and local neural circuits.

The physiologic mechanism of IHI measured by TMS has not been fully understood. For the early component with the ISI of ~ 10 ms (IHI10) where we found the tendency of reduction after tSMS, the transcallosal connection between bilateral homologous regions might be a candidate because the direct conduction time has been estimated to be ~ 10 ms^39, 40^. For IHI40 which was found to be significantly decreased after tSMS, the exact pathway responsible for this inhibition is not known yet, but may include multi-synaptic pathways including association cortex, thalamus or basal ganglia and/or recurrent connections^41–43^. It was suggested that different neuronal population might mediate short and long latency IHI^44^. In terms of neurotransmitter systems, previous studies showed the LIHI at ISIs of 20–50 ms was mainly mediated via postsynaptic GABAB receptors in human study^43, 45^. In vivo study also showed that GABAB receptors in the layer V of the cortex mediated the IHI in the cellular level^46^. The reduction of IHI40 in the present study indicates the possibility that the PFF of the right M1 is partly related to the large brain network including both M1 rather than the direct transcallosal pathway.

Since tSMS can reduce the corticospinal excitability of the stimulated (left) M1, it is possible that the power of inhibition within the right M1 might be reduced just because the conditioning pulse over the left M1 recruits a reduced neuronal pool targeting the right M1. At least, there was no significant correlation between the tSMS-induced reduction of the corticospinal excitability of the left M1 and the reduction of IHI measured at the right M1. However, neural populations with corticospinal output and those with transcallosal output are different, suggesting the non-linear relationship between them. Thus, further animal studies might be necessary to investigate the IHI modulation in detail.

Since we only estimated the modulation of IHI from the left to the right M1, it is possible that the IHI of the inverse direction might behave differently when tSMS was given over the right M1. Further studies about the lateralized differences in efficacy of tSMS might be needed in the future, because it is still in debate whether the laterality of IHI strength exists or not^47–49^.

NIBS has been used for the rehabilitation of the motor and nonmotor function after stroke^50–53^, including the clinical application of PFF principle. For example, the low-frequency rTMS has been used over the healthy hemisphere to suppress the IHI, which improved the motor function^10, 54^, aphasia^55^ and unilateral special neglect^56, 57^. Therefore, we expect that tSMS can produce a positive effect on stroke patients based on the PPF effect shown in our study. The magnet used for tSMS is not expensive and easy to handle compared to rTMS, which might facilitate the future clinical use for stroke patients.

Our findings provide new insights on the mechanistic understanding of neuromodulatory effects of tSMS in human and suggest the possible clinical application to facilitate the affected functions in stroke and other neurodegenerative diseases.

## Methods

### Subjects

This study involved two experiments. The first experiment (Expt. 1) examined the effects of tSMS on the cortical excitability in both M1, and nineteen healthy subjects (nine males and ten females; aged 21–38; mean age, 26 ± 4 years) participated. The second experiment (Expt. 2) examined the effects of tSMS on the IHI from the left to the right M1, and eighteen healthy subjects (eight males and ten females; aged 21–39; mean age, 26 ± 5 years) participated. No subjects had a history of neurological illness by self-report. All subjects were right-handed according to Oldfield’s handedness inventory^58^. This study was approved by the Ethics Committee of Ritsumeikan University (Kyoto, Japan) and Murata Hospital (Osaka, Japan), and all experiments was performed in accordance with the guidelines by The Safety of TMS Consensus Group^59^ and the regulations in each institution. All participants gave their written informed consent.

### Measurement of brain function by TMS

We measured the brain function by TMS technique using a flat figure-of-eight coil (Magstim 200, The Magstim Company Ltd.). The handle of the coil pointed backwards (approximately 45° from the midsagittal line) and the direction of induced current in the brain was from posterolateral to anteromedial^24^.

The electromyogram (EMG) was recorded from the right and left FDI muscles. The EMG signals were amplified, band-pass-filtered (5–1000 Hz), and digitized at a rate of 10 kHz using the Map1200 system (Nihon-Santeku Co., Ltd., Osaka, Japan).

For the evaluation of the corticospinal excitability (Expt.1), single-pulse TMS was used. The rMT for FDI muscle was defined as the minimum TMS intensity required to induce a MEPs of > 50 μV peak-to-peak amplitude in at least five of ten trials^24, 60^. The peak-to-peak MEPs amplitudes of the FDI muscle at least ten trials were measured, and the averages were taken. The intensity of the test stimulus was adjusted to produce an MEPs of ~ 1 mV from the target FDI muscle before the intervention (SI1mV).

For the evaluation of the IHI (Expt. 2), paired-pulse TMS over each hemisphere was used. The first TMS over the left M1 (the conditioning stimulus, 120% of the rMT) was followed by the second TMS over the right M1 (the targeted side, SI1mV) with the interstimulus interval (ISI) of 10 or 40 ms (IHI10, IHI40). Test stimuli (the right M1 only) without conditioning stimuli and paired ones (IHI10, IHI40) were presented randomly. At least ten trials were recorded from the left FDI and averaged for each condition. The IHI ratio was computed by dividing the paired MEPs by the single (test) MEPs amplitude, separately for IHI10 and IHI40.

### Experimental procedures

For tSMS, we used a cylindrical neodymium (NdFeB) magnet of 50-mm diameter and 30-mm thickness (Model N50, NeoMag Co., Ltd., Chiba, Japan). The surface magnetic flux density was 5340 G and nominal strength was 88 kgf. A non-magnetic stainless-steel cylinder of the same size was used for sham stimulation. The NdFeb magnet and non-magnet were set by using an arm-type light stand (C-stand, Avenger, Cassola, Italy) over the representational area for the right FDI muscle identified by TMS. The intervention duration was 30 min. We fixed the polarity of magnet to the north side, because the previous study reported the indifference of polarity for the neural effects^13^.

The subjects were seated in a reclining chair that allowed them to keep relaxed during TMS measurement and magnetic or sham stimulation in Expts. 1 and 2. For Expt. 1, we measured MEPs and rMT for both FDI muscles at before (Pre), immediately after (Post-0), 10 min after (Post-10) and 30 min after (Post-30) the intervention. EMG was recorded from the right FDI muscle by stimulating the left M1 and from the left FDI muscle by stimulating the right M1. The order of the measurement, right or left hand, was pseudo-randomly determined to be counterbalanced. The average measurement completion time at each point was less than 5 min.

For Expt. 2, after locating the hot spots and measuring rMT of both FDI muscles, we measured the IHI before (pre) and immediately after (post) the intervention.

The design of the two experiments was double-blind crossover trial, and the interval of real and sham stimulation was at least 3 days. The order of conditions was pseudo-randomly determined to be counterbalanced.

### Statistical analysis

For Expt. 1, three-way repeated-measures ANOVA was used with Time (Pre, Post-0, Post-10, Post-30), Hands (right, left) and Condition (real, sham) as within-subject factor. For Expt. 2., three-way repeated-measures ANOVA was used Time (Pre, Post), ISI (IHI10, IHI40) and Condition (real, sham) as within-subject factor. The Greenhouse–Geisser method was used to adjust for sphericity if needed. In case of significant interaction effects, post hoc analyses were performed by Student’s paired-samples t-test with a Bonferroni correction for multiple comparisons. To investigate whether the reduction of IHI is affected by the excitability reduction of the left M1 in a non-parametric way, Spearman’s rho was computed for the ratio between the MEP amplitudes before and after intervention and the ratio between the IHI before and after intervention for the real condition. SPSS ver.23 statistical software was used to analyze the data and the criterion for significance was set at p < 0.05. All data are shown as mean ± SEM.
